# *Lactobacillus* protects the integrity of intestinal epithelial barrier damaged by pathogenic bacteria

**DOI:** 10.3389/fcimb.2015.00026

**Published:** 2015-03-25

**Authors:** Qinghua Yu, Lixia Yuan, Jun Deng, Qian Yang

**Affiliations:** College of Veterinary Medicine, Nanjing Agricultural UniversityNanjing, China

**Keywords:** *Lactobacillus*, paracellular permeability, IL-8, mucosal barrier, tight junction

## Abstract

Pathogens invade intestinal mucosal barrier through phagocytosis of antigen presenting cells (dendritic cell, microfold cells), or through the invasion into the intestinal epithelial directly. Some pathogens could damage the cell junction between epithelial cells and use the paracellular pathway as an entrance to invade. Moreover, some *Lactobacillus* could inhibit the adhesion of the pathogens and protect the integrity of the cell junction and mucosal barrier. This research focused on the potential therapeutic effect of *Lactobacillus fructosus* (*L. fructosus*) C2 to attenuate ETEC K88 or *S. typhimurium* SL1344 induced changes to mucosal barrier. The results demonstrated that treatment of polarized Caco-2 cells with *L. fructosus* C2 reduced the permeation of dextran, and expression of IL-8, p-ERK, and p-JNK when cells were infected with pathogenic bacteria. The findings indicated that *L. fructosus* C2 exerted a protective effect against the damage to the integrity of Caco-2 cells by ETEC or *S. typhimurium* infection.

## Introduction

The intestinal epithelium constitutes the major barrier that separates the internal from the external environment, providing high resistance to free diffusion of solutes and entry of pathogens and harmful antigens. In order to establish an efficient barrier, intercellular spaces must be strictly sealed by cell junctions, which constitute continuous circumferential seals around cells. Several junction proteins have been identified in this region. For example, occludin and claudins are integral membrane proteins believed to associate with cytoplasmic plaque proteins such as ZO-1 (Muza-Moons et al., 2004). However, several pathogens, such as *Salmonella enterica* serovar Typhimurium (*S. typhimurium*) and enterotoxigenic *Escherichia coli* (ETEC), could adhere to the brush border of intestinal cells, damage the structure of tight junctions or adherens junction (Blum and Schiffrin, 2003; de Vrese and Marteau, 2007). *S. typhimurium* and ETEC could also cause inflammation and IL-8 expression from the basal side of infected intestinal cells, directing neutrophil transmigration. IL-8 expression by infected intestinal epithelial cells is regulated via various signaling pathways, such as elevation of intracellular Ca^2+^ (Riederer et al., 2011), mitogen-activated protein (MAP) kinases (Mizuno et al., 2011), protein kinase C (PKC) (Riederer et al., 2011), protein kinase A (PKA) (Riederer et al., 2011). Recent publications showed that specific enzymes in the extracellular signal-regulated kinase (ERK) and c-Jun N-terminal kinase (JNK) pathway could directly activate the NF-κB regulatory cascade, regulating tight junctions (Terakado et al., 2011; Petecchia et al., 2012).

Prevention of *S. typhimurium* and ETEC infection are essential to the public health. Antibiotics and vaccines were both used to fight pathogens but they have limitation, causing drug resistance and providing inefficient protection because of many serotypes of bacteria. Recently, increasing evidences showed that lactobacilli are effective in preventing intestinal disease in both humans and animals due to their ability to maintain or restore normal microbiota, inhibit pathogen adhesion to the intestinal mucosa, and prevent inflammatory processes (Blum and Schiffrin, 2003; Sartor, 2005; de Vrese and Marteau, 2007; Zhou et al., 2010).

The physiological consequences of *Lactobacillus* strains and pathogenic bacteria induced alteration of tight junctions and adherens junction function needed to be addressed *in vitro* (cell models). Caco-2 cells, a model of mature enterocytes of the intestine, have been widely used for the research of the interaction between bacteria and intestinal epithelium (Fang et al., 2014; Finamore et al., 2014). In this study, we hypothesized that treatment of epithelial cells with *L. fructosus* C2 may protect them from the deleterious effects of subsequent infection with *S. typhimurium* SL1344 or ETEC K88, and sustain the cell integrity. The aim of this study therefore was to investigate whether *L. fructosus* C2 was able to protect against intestinal injury induced by ETEC or *S. typhimurium* infection and the related signal pathways. We hoped to provide a rationale for the use of probiotics as therapeutic agents and reveal the effects of *Lactobacillus* strains on cell integrity and intestine barrier.

## Materials and methods

### Bacterial growth

ETEC strain K88 and *S. typhimurium* SL1344 were grown in Luria-Bertani (LB) broth. Lactobacillus fructosus C2, isolated from chicken intestine, could inhibit enteric pathogenic bacteria adhesion on Caco-2 cells (Yu et al., 2007, 2011). *L. fructosus* C2 was selected and grown in DeMan Rogosa Sharp (MRS) medium at 37°C under anaerobic conditions. Bacterial concentrations of ETEC, *S. typhimurium* SL1344, and *L. fructosus* C2 were confirmed by serial dilutions followed by CFU counts. Bacteria were harvested by centrifugation and then resuspended in antibiotic-free DMEM medium. The viability of bacteria grown on DMEM did not differ from that of bacteria grown on LB or MRS media.

### Cell culture

The cancer cell Caco-2 line, a model of mature enterocytes, was maintained in DMEM supplemented with 10% fetal bovine serum. The Caco-2 cells were trypsinized, washed and resuspended, and then seeded on the Millicell filter inserts (Millipore, USA) at a seeding density of 10^4^ cells/cm^2^. Cells reached confluence and were used for experimentation between days 14 and 21. The cell culture medium was changed to fresh medium without antibiotics prior to treatment of the cells with bacteria.

### Dextran permeability as measurements of changes in the barrier function of polarized epithelial cell monolayers

As previously described (Johnson-Henry et al., 2008), polarized Caco-2 cells were grown on Millicell filter inserts and cultured until the transepithelial electrical resistance (TER) reached a minimum of 1000 Ω cm^2^. The cells were treated with 1 mL of medium containing pathogens (ETEC K88 or *S. typhimurium* SL1344) (MOI 20:1) and *L. fructosus* C2 (MOI 200:1) either alone or simultaneously for 2 h. The movement of macromolecules across polarized epithelial cell monolayers was assayed using a macromolecular conjugate probe, fluorescein-isothiocyanate dextran of molecular masses of 70 kDa (FD 70S) (Sigma). Briefly, 0.2 mL of conjugated dextran (final concentration, 1 mg/mL) suspended in DMEM was added to the apical compartment of Millicell filter inserts, and 0.4 mL of DMEM alone added to the basolateral compartment. After incubation for 2 h at 37°C, samples (0.1 mL) from the apical and basolateral compartment were placed into a 96 well plate and analyzed to determine their fluorescent intensity using the bio-tek fluorescence spectrophotometer.

### Measurement of TER and IL-8 assay

Caco-2 cells were grown until a TER of at least 1000 Ω cm^2^ was achieved. *L. fructosus* C2 (MOI 200:1) or pathogenic bacteria (ETEC or *S. typhimurium* SL1344, MOI 20:1) were diluted in DMEM without antibiotic and added to the apical compartment of Millicell filter inserts either alone or simultaneously. Control Caco-2 cells were cultured only with DMEM.

TER was employed as a marker of intercellular tight junction integrity, because it provides an electrical measurement of barrier function toward passive ion flow and the measurements are inversely related to the permeability of a polarized epithelium to macromolecules. The TER assay was performed on the Caco-2 cell monolayers at 0, 1, 2, 3, 4, 6, and 12 h by measuring the TER using a Millicell-ERS-II Volt Ohm meter (Millipore). Meanwhile, after incubation for 6 h, the basolateral medium was collected and used for IL-8 assay. The IL-8 concentration in the basolateral medium was determined with the human IL-8 ELISA kit (Boster, Wuhan, China). The experiments were repeated with four times.

### The change of tight junction by transmission electron microscopic and fluorescence microscopy

After the pathogenic bacteria (ETEC or *S. typhimurium* SL1344) or *L. fructosus* C2 treatment, the medium was removed, and the membranes were excised and fixed in 0.1 M phosphate buffer containing 2.5% glutaraldehyde and 2.0% paraformaldehyde (pH 7.4). The membranes were washed three times in 0.1 M phosphate buffer for 20 min each. The membranes were then fixed in 2% osmium tetroxide and dehydrated in increasing concentrations of acetone. Dehydrated membranes were subsequently infiltrated with epoxy resin, embedded in silicone molds, polymerized and then examined using the transmission electron microscope.

The immunofluorescence staining protocol was adapted from the protocol (Zareie et al., 2005). Briefly, confluent Caco-2 cell monolayers were rinsed in PBS, and followed by fixation and permeabilization in 5% formaldehyde 20°C for 15 min. Then Caco-2 cells were incubated in 5% (vol/vol) normal goat serum in PBS for 1 h at room temperature and then incubated with primary antibodies against occludin (Zymed, San Francisco, CA) at 37°C overnight. After unbound primary antibodies were rinsed away with PBS, cells or tissues were incubated with secondary FITC conjugated goat anti-mouse IgG (1:500 dilution; Boster) for 1 h at room temperature. Caco-2 cells were thoroughly rinsed with PBS and examined with a fluorescence microscope (Olympus BX5, Japan).

### Analysis of ERK, JNK expression on Caco-2 cells with western blotting

Caco-2 cell monolayers were collected immediately snap-frozen in liquid nitrogen. In preparation for SDS-PAGE, cells were thawed to 4°C and homogenized in chilled RIPA buffer, including protease and phosphotase inhibitors. After centrifugation at 10, 000 × g for 10 min at 4°C, the supernatant was recovered and assayed for protein concentrations determined by Bradford method.

Equal amounts of total protein were separated on 10% SDS-polyacrylamide gels and then transferred to the PVDF membrane. After blocking over night, membranes were washed three times and incubated with antibodies specific for phosphorylated forms of ERK1/2, or JNK1/2/3, or total ERK1/2, or total JNK1/2/3 for over night at 4°C. The membranes were then incubated with horseradish peroxidase-conjugated secondary antibody. Following two washes with TBST and one wash with TBS, the membranes were developed for visualization of protein by the addition of enhanced chemiluminescence reagent. The mean density of the phosphorylated ERK or JNK band was divided by the mean density of the corresponding total ERK or JNK band to yield a normalized band density value.

### Statistics

The results were expressed as means ± standard errors of the means. The one-way analysis of variance (ANOVA) is used to determine statistical significant differences between multiple groups. A *P*-value < 0.05 denoted statistically significant differences.

## Results

### *L. fructosus* C2 reduced dextran permeability

**Figure 1:**
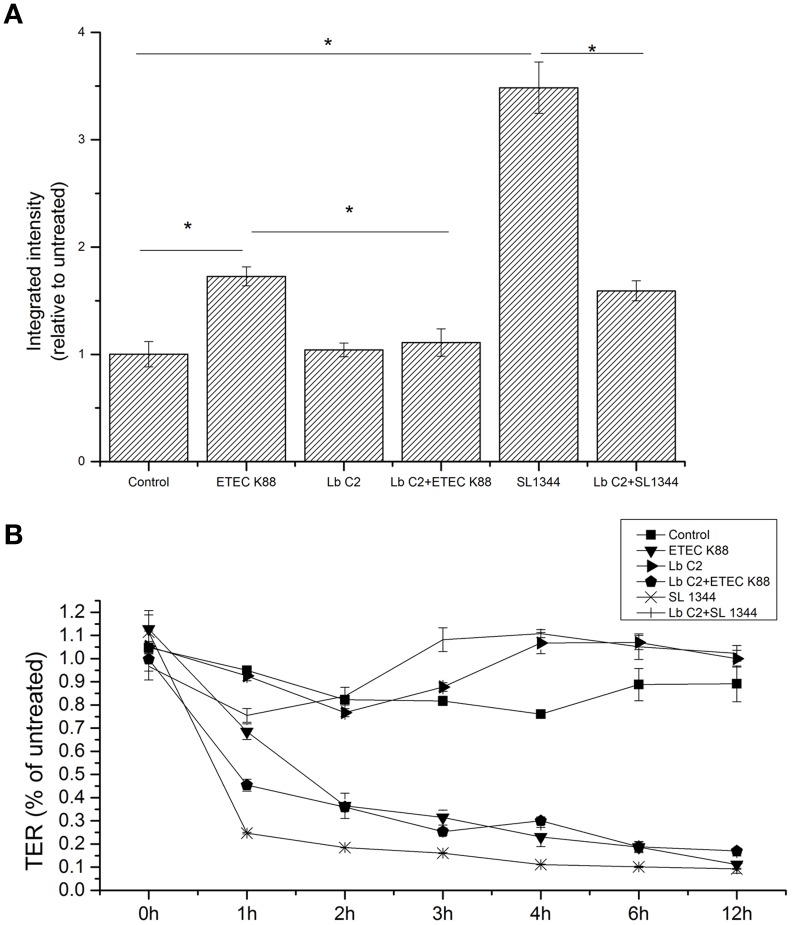
***L. fructosus* C2 decreased pathogenic bacteria induced increases in macromolecular permeability (A) and TER (B)**. Polarized monolayers were treated with *L. fructosus* C2 (MOI 200:1) or pathogens (ETEC or *S. typhimurium*, MOI 20:1) either alone or simultaneously for 2 h. Integrated intensities of dextran diffused into the basolateral compartments were detected 2 h after dextran addition to the apical compartment. TER values were detected at 0, 1, 2, 3, 4, 6, and 12 h post co-infection. ^*^P < 0.05, as determined by ANOVA. The data represent results from four independent experiments.

The results demonstrated that treatment with *L. fructosus* C2 alone did not alter the diffusion of the FD70s from apical to basolateral Millicell compartments [relative integrated intensity (RI) compared to untreated monolayers] (Figure 1A). However, compared to the control group, ETEC K88 or *S. typhimurium* SL1344 infection did exhibit a remarkable increase in the permeability to the dextran probe (*P* < 0.05). Treatment of Caco-2 cells with *L. fructosus* C2 and pathogens (ETEC K88 or *S. typhimurium* SL1344) simultaneously, the permeability to the dextran probe was decreased significantly (*P* < 0.05). These results revealed that *L. fructosus* C2 preserved intestinal epithelial cell integrity.

### The effect of *L. fructosus* C2 on the TER

Treatment of Caco-2 cells with *L. fructosus* C2 alone did not alter TER value at 1 h and maintain the stable state within 12 h (Figure 1B). As observed, ETEC K88 or *S. typhimurium* SL1344 infection reduced TER of Caco-2 cells significantly (*P* < 0.05) at 12 h. However, treatment of polarized monolayers with *L. fructosus* C2 significantly attenuated the decrease in TER induced by *S. typhimurium* SL1344 (*P* < 0.05). *L. fructosus* C2 did not attenuate ETEC-induced drop in TER. Moreover, *L. fructosus* C2 significantly potentiated ETEC-induced drop in TER at 1 h.

### *L. fructosus* C2 counteracted the *E. coli* and *S. typhimurium* induced dysregulation of IL-8 expression

**Figure 2:**
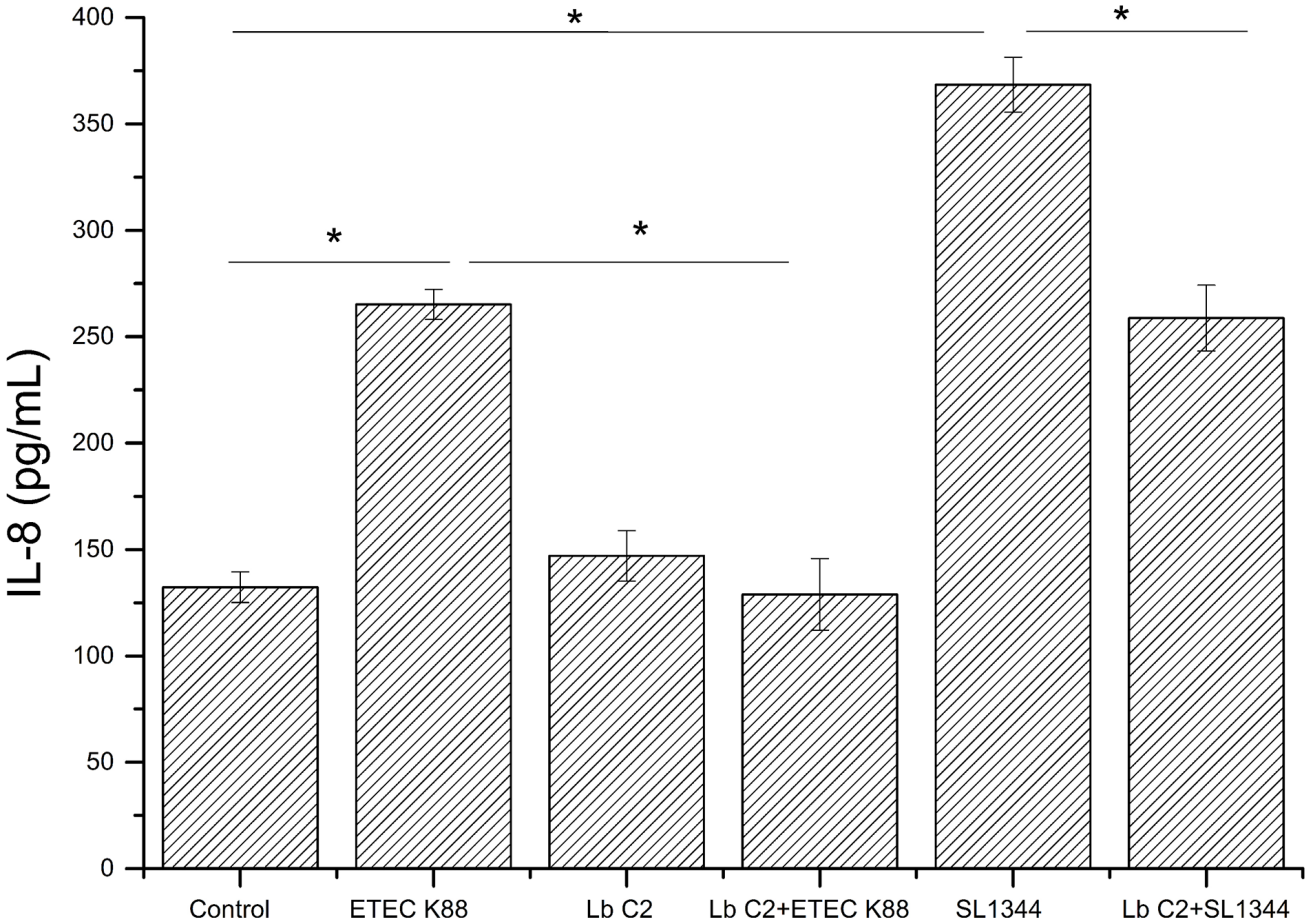
**Effects of *L. fructosus* C2 on IL-8 production**. IL-8 production was measured by ELISA method after cells were treated with *L. fructosus* C2 (MOI 200:1) or pathogens (ETEC or *S. typhimurium*, MOI 20:1) simultaneously for 6 h. ^*^P < 0.05, as determined by ANOVA. The data represent results from four independent experiments.

The IL-8 expression was evaluated, when the Caco-2 cells were co-cultured with pathogens (ETEC K88 or *S. typhimurium* SL1344), *L. fructosus* C2 alone or simultaneously (Figure 2). Infection with ETEC K88 or *S. typhimurium* SL1344 caused a significant up-regulation of IL-8 expression compared to the untreated cells (*P* < 0.05). However, we found that treatment with *L. fructosus* C2 and ETEC K88 simultaneously inhibited the increase of IL-8 expression caused by ETEC alone significantly (*P* < 0.05). Compared to the *S. typhimurium* SL1344 infection, co-culture with *L. fructosus* C2 and *S. typhimurium* SL1344 could reduce the IL-8 secretion significantly, but still could not recover to the normal status.

### *L. fructosus* C2 prevented ETEC K88 and *S. typhimurium* SL1344 induced ultrastructural changes in polarized epithelial monolayers

**Figure 3:**
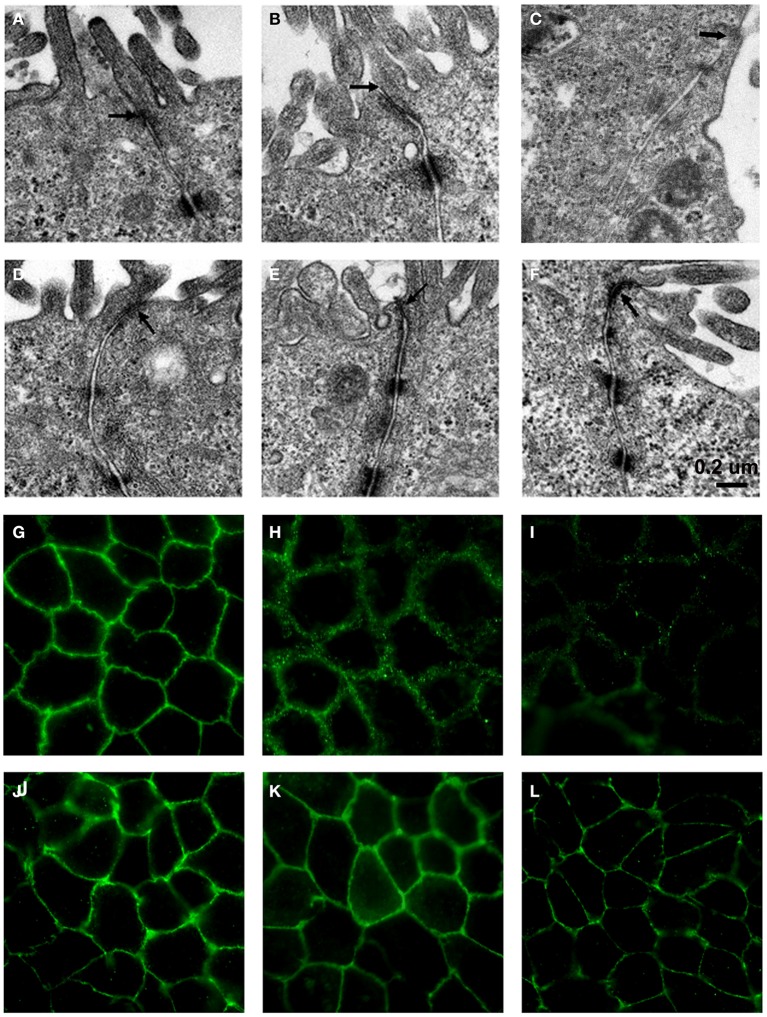
***L. fructosus* C2 inhibited ETEC K88 or *S. typhimurium* SL1344 induced tight junction changes of Caco-2 cells**. Polarized monolayers were treated with *L. fructosus* C2 (MOI 200:1) or pathogens (ETEC or *S. typhimurium*, MOI 20:1) either alone or simultaneously for 2 h. **(A,G)** cells without treatment. **(B,H)** Cells treated with ETEC K88. **(C,I)** Cells treated with *S. typhimurium* SL1344. **(D,J)** Cells treated with *L. fructosus* C2. **(E,K)** Cells treated with *L. fructosus* C2 and ETEC K88 simultaneously. **(F,L)** Cells treated with *L. fructosus* C2 and *S. typhimurium* SL1344 simultaneously. Arrow showed the tight junction.

Transmission electron microscopy of untreated Caco-2 epithelial cells revealed the tight junctions located at the apical side of cell monolayers, appear as sites where the intercellular space between neighboring cells is obliterated and the adjoining membranes appear to fuse (Figure 3A). Adherens junction and desmosomes were also visible below the tight junction. In contrast, in Caco-2 cells infected with ETEC K88 or *S. typhimurium* SL1344, the tight junction became wider (Figures 3B,C). When treated with *L. fructosus* C2 alone, Caco-2 cells presented a normal brush border and without distinct ultrastructural changes in tight junctions morphology (Figure 3D). Cells treated with *L. fructosus* C2 and ETEC K88 or *S. typhimurium* SL1344 simultaneously showed membrane disruption to a lesser extent compared with the infected group (Figures 3E,F).

The protection effect of *L. fructosus* C2 on tight junction was also verified by immunofluorescence staining of occludin. Caco-2 cells untreated or treated with *Lactobacillus fructosus* C2 alone had intact cell junctions, as demonstrated by continuous and circumferential occludin distribution visualized by fluorescence microscopy. In contrast, discontinuous fluorescence lines in the cell membrane and cytoplasm labeling the proteins were observed in cells infected with ETEC K88 or *S. typhimurium* SL1344. While Caco-2 cells were co-cultured with *Lactobacillus fructosus* C2 and pathogens simultaneously, the distribution of green spots was increased along the cell border as compared to the ETEC or SL1344 infection group, indicating that the changes of occludin redistribution caused by ETEC or SL1344 were ameliorated (Figure 3).

### The influences of *L. fructosus* C2 on the ERK and JNK

**Figure 4:**
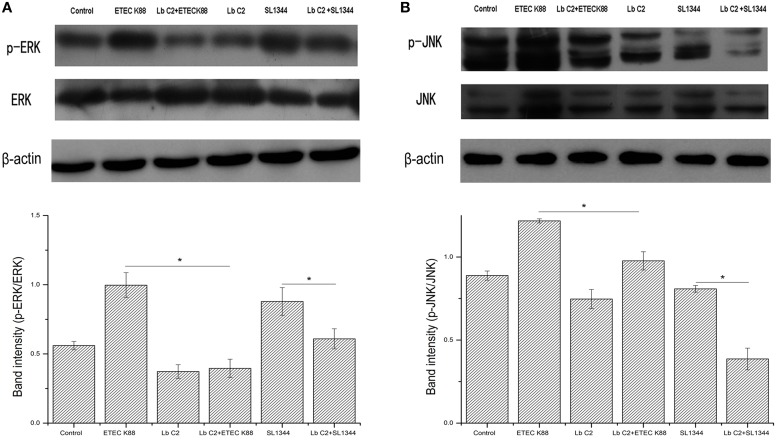
***L. fructosus* C2 inhibited the phosphorylation of ERK (A) and JNK (B) caused by ETEC or *S. typhimurium* on Caco-2 cells**. Caco-2 cells were untreated (Control), infected with ETEC or *S. typhimurium* (MOI 20:1) alone or co-cultured with *L. fructosus* C2 (MOI 200:1) simultaneously for 2 h. The figure showed a western blot of immunoprecipitated ERK and JNK level, representative of four independent assays, and the densitometric values of phosphorylation of ERK and JNK normalized to total ERK and JNK. Values are means ± SE. ^*^P < 0.05, as determined by ANOVA.

We next investigated the activation of ERK and JNK in Caco-2 cells treated with *L. fructosus* C2 alone or with pathogens (ETEC or *S. typhimurium*) simultaneously. The results demonstrated that the phosphorylation level of ERK and JNK were increased significantly while infected with ETEC K88 alone (*P* < 0.05) (Figure 4). However, the phosphorylation level of ERK and JNK were reduced significantly co-cultured with *L. fructosus* C2 and pathogens simultaneously, compared to the pathogens infected alone.

## Discussion

Pathogens, including EPEC, enterotoxigenic *E. coli* (ETEC) and enterohaemorrhagic *E. coli* (EHEC), may activate myosin light chain kinase leading to contraction of the circumferential actomyosin ring, thus causing damage to cell junctions and influencing the permeability (Philpott et al., 1998; Johnson et al., 2010). Pathogens can also secrete toxins that target tight junction proteins including *Clostridium perfringes* enterotoxin (CPE), *Bacteroides fragilis* toxin and *Vibrio cholerae* HA/P, leading to disruption of the cell junction complexes and decreased barrier function. This study broadens our current understanding of how probiotics exert their beneficial effects and emphasize the ability of *L. fructosus* C2 to protect polarized epithelial cells against the effects of ETEC or *S. typhimurium* SL1344 -induced changes in barrier function at several different levels. The results demonstrated that treatment with *L. fructosus* C2 attenuated the pathogen-induced alterations in epithelial barrier function. This study demonstrated that infection with ETEC K88 or *S. typhimurium* SL1344 resulted in decreased TER and increased permeability to FD70S. *L. fructosus* C2 alone would not influence the integrity of cell barrier and ameliorate the changes of TER and permeability to FD70S. The results implied that *L. fructosus* C2 could protect the epithelial barrier and maintain the integrity of cell junctions, which was correspond to previous study (Anderson et al., 2010).

Many cytokines have been shown to regulate the cell junctions and cytoskeleton structure and function (Nusrat et al., 2000). IL-8, a well-known proinflammatory cytokine inducer recruiting neutrophils to phagocytose the antigen, has been associated with pathogen-induced alterations of tight junction (Otte and Podolsky, 2004). In agreement with these findings, we found that disruption of the membrane barrier by ETEC or *S. typhimurium* was associated with a strong increase of IL-8 expression. However, treatment with *L. fructosus* C2 would reduce the IL-8 expression, suggesting *L. fructosus* C2 could prohibit the occurrence of inflammation.

Mitogen-activated protein (MAP) kinases signaling pathway is able to modulate paracellular transport by up or down regulating the expression of cell junction proteins and hence altering the molecular composition (Gonzalez-Mariscal et al., 2008). The mechanisms by which probiotics exert their beneficial effects on epithelial barrier function remain unclear. Several studies have shown that probiotics could inhibit epithelial barrier disruption by MAPK dependent mechanisms (Eun et al., 2011; Segawa et al., 2011). In the experiments, we found that ETEC K88 increased the phosphorylation level of ERK, JNK and altered the tight junctions, which were correspond with the increase permeation of FD70s and the increase of TER. Activation of ERK1/2 by transfection with an activated Ras mutant increases six fold the transepithelial permeability of mannitol and more than 40% the TER (Gonzalez-Mariscal et al., 2008). *L. casei* could protect the intestinal epithelial and suggest that the MAPK signaling pathway may in part mediate the effects of probiotics on the intestinal epithelial barrier (Eun et al., 2011).

Although many clinical studies have reported that *Lactobacillus* strains exert beneficial health effects by producing bacteriostatic or bactericidal agents (Takahashi et al., 2004; Corr et al., 2007; Kumar et al., 2011), competitively excluding pathogenic bacteria (Sherman et al., 2005) or regulating immunomodulatory effects (Resta-Lenert and Barrett, 2006), it is still difficult to ascertain their direct mechanisms of action. The results reported here indicate that *Lactobacillus fructosus* C2 may prevent the membrane barrier disruption caused by the pathogens. The maintenance of barrier integrity was achieved by increase of TER, downregulation of permeability of FD70S and IL-8, regulation of ERK and JNK. These results provide new insights into the protective activity of lactobacilli, supporting the view that they may act through diverse mechanisms.

## Author contributions

QY: Substantial contributions to the conception and perform the experiment. LY, JD: sample collection and analysis. QY: Substantial contributions to the conception or design of the work.

### Conflict of interest statement

The authors declare that the research was conducted in the absence of any commercial or financial relationships that could be construed as a potential conflict of interest.
