# Corrigendum: *Lactobacillus* protects the integrity of intestinal epithelial barrier damaged by pathogenic bacteria

**DOI:** 10.3389/fcimb.2020.585198

**Published:** 2020-10-29

**Authors:** Qinghua Yu, Lixia Yuan, Jun Deng, Qian Yang

**Affiliations:** College of Veterinary Medicine, Nanjing Agricultural University, Nanjing, China

**Keywords:** *Lactobacillus*, paracellular permeability, IL-8, mucosal barrier, tight junction

In the original article, there was a mistake in Figure 3. The blank control (Figures 3A,G), Figures 3H,I were mistakenly presented with incorrect images. The corrected Figure 3 appears below.

**Figure 3:**
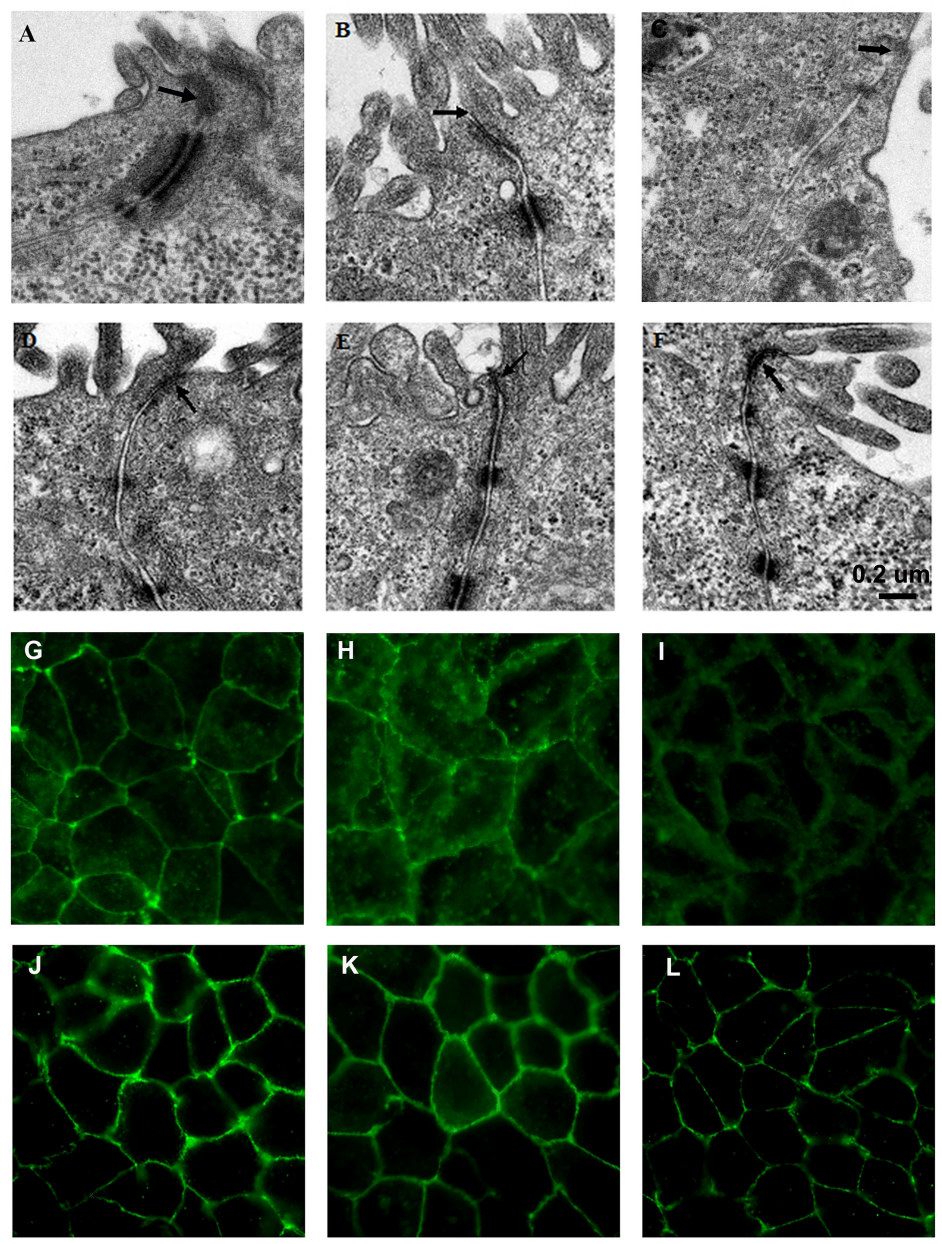
*L. fructosus* C2 inhibited ETEC K88 or *S. enterica serovar Typhimurium* SL1344 induced tight junction changes of Caco-2 cells. Polarized monolayers were treated with *L. fructosus* C2 (MOI 200:1) or pathogens (ETEC or *S. enterica serovar Typhimurium*, MOI 20:1) either alone or simultaneously for 2 h. **(A,G)** cells without treatment. **(B,H)** Cells treated with ETEC K88. **(C,I)** Cells treated with *S. enterica serovar Typhimurium* SL1344. **(D,J)** Cells treated with *L. fructosus* C2. **(E,K)** Cells treated with *L. fructosus* C2 and ETEC K88 simultaneously. **(F,L)** Cells treated with *L. fructosus* C2 and *S. enterica serovar Typhimurium* SL1344 simultaneously. Arrow showed the tight junction.

The authors apologize for this error and state that this does not change the scientific conclusions of the article in any way. The original article has been updated.

